# The innovative checkpoint inhibitors of lung adenocarcinoma, cg09897064 methylation and ZBP1 expression reduction, have implications for macrophage polarization and tumor growth in lung cancer

**DOI:** 10.1186/s12967-024-04995-1

**Published:** 2024-02-18

**Authors:** Ailing Wang, Wei-sha Zheng, Zhen Luo, Lian Bai, Shi Zhang

**Affiliations:** 1 Department of Pulmonary and Critical Care Medicine, Central Hospital Affiliated to Shandong First Medical University, Jinan, China; 2 Jiangsu Provincial Key Laboratory of Critical Care Medicine, Department of Critical Care Medicine, School of Medicine, Zhongda Hospital, Southeast University, Nanjing, Jiangsu China; 3 Central Hospital Affiliated to Shandong First Medical University, Jinan, China

## Abstract

Lung cancer, a prevalent and aggressive disease, is characterized by recurrence and drug resistance. It is essential to comprehend the fundamental processes and discover novel therapeutic objectives for augmenting treatment results. Based on our research findings, we have identified a correlation between methylation of cg09897064 and decreased expression of ZBP1, indicating a link to unfavorable prognosis in patients with lung cancer. Furthermore, these factors play a role in macrophage polarization, with ZBP1 upregulated in M1 macrophages compared to both M0 and M2 polarized macrophages. We observed cg09897064 methylation in M2 polarization, but not in M0 and M1 polarized macrophages. ATACseq analysis revealed closed chromatin accessibility of ZBP1 in M0 polarized macrophages, while open accessibility was observed in both M1 and M2 polarized macrophages. Our findings suggest that ZBP1 is downregulated in M0 polarized macrophages due to closed chromatin accessibility and downregulated in M2 polarized macrophages due to cg09897064 methylation. Further investigations manipulating cg09897064 methylation and ZBP1 expression through overexpression plasmids and shRNAs provided evidence for their role in modulating macrophage polarization and tumor growth. ZBP1 inhibits M2 polarization and suppresses tumor growth, while cg09897064 methylation promotes M2 polarization and macrophage-induced tumor growth. In mechanism investigations, we found that cg09897064 methylation impairs CEBPA binding to the ZBP1 promoter, leading to decreased ZBP1 expression. Clinical experiments were conducted to validate the correlation between methylation at cg09897064, ZBP1 expression, and macrophage M2 polarization. Targeting these factors may hold promise as a strategy for developing innovative checkpoint inhibitors in lung cancer treatment.

**Supplementary Information:**

The online version contains supplementary material available at 10.1186/s12967-024-04995-1.

## Introduction

The Global Cancer Report reveals that lung cancer is a widespread malignant tumor, with a mortality rate of 82–89% worldwide. In China, 730,000 new cases and 610,000 deaths are reported annually, with a 5-year survival rate of 11–17%- a grave health hazard and social burden [1–3].Of all the lung cancer types, adenocarcinoma is the most prevalent, accounting for more than 50%of cases [4, 5].

In the past decade, lung cancer treatment has seen advances in early detection, targeted therapy, and immunotherapy, which have extended patient survival. However, drug resistance and recurrence remain issues [5]. Consequently, it is essential to investigate multiple treatment options in order to tackle these problems and progress lung cancer treatment.

The development of lung cancer is profoundly impacted by epigenetic alterations, both genetically and environmental. Epigenetic factors, such as abnormal DNA methylation induced by risk factors like smoking and chronic lung disease, can deactivate tumor suppressor genes and activate oncogenes. This process ultimately leads to the development of lung cancer. Furthermore, site-specific methylation may contribute to treatment resistance, making epigenetic changes promising therapeutic targets for lung cancer [6–8].

The understanding of tumor immune infiltration's molecular mechanisms has led to the development of multiple immune checkpoints and targeted drugs. This progress has made immunotherapy a significant treatment modality for lung cancer. Monoclonal antibodies targeting PD-1/PD-L1 have been extensively utilized in cancer treatment [9, 10]. However, challenges such as drug resistance, recurrence, and tumor heterogeneity still hinder the efficacy of immunotherapy. Further investigation is necessary to expand our understanding of immune infiltration processes in lung cancer. This will help in the development of new immune checkpoints and complementary immune-targeted therapies, ultimately improving treatment outcomes and patients' quality of life. Epigenetic regulation may possibly augment immune infiltration [9, 10]. Currently, immune therapy primarily targets downstream immune co-stimulatory protein molecules. By regulating epigenetics upstream and blocking immune checkpoint transcription, immune infiltration can be fundamentally improved. Epigenetic drugs [11, 12] primarily target DNA methylation, the most prevalent epigenetic alteration.

This study examines the mechanistic association between cg09897064 methylation on Z-DNA binding protein 1 (ZBP1) in lung adenocarcinoma. It explores the implications for patients' prognosis, macrophage polarization, and tumor growth. Our results demonstrate that cg09897064 methylation leads to a decrease in ZBP1 expression. This, in turn, causes macrophages to polarize towards the M2 phenotype and promotes tumor growth. Mechanistically, we discovered that cg09897064 methylation impairs CCAAT Enhancer Binding Protein Alpha (CEBPA) binding to the ZBP1 promoter, resulting in decreased ZBP1 expression. Clinical experiments were conducted to validate the correlation between methylation at cg09897064, ZBP1 expression, and macrophage M2 polarization. The potential for the invention of pioneering checkpoint inhibitors in lung cancer treatment is great if these factors are targeted.

## Materials and methods

### High-throughput data analysis

Data of lung adenocarcinoma and its neighboring normal tissues' transcript some and DNA methylation were procured from The Cancer Genome Atlas(TCGA)database. In the TCGA database, a comprehensive search was conducted for all expression microarray datasets associated with lung adenocarcinoma up until January 2022. Animal and cell model data related to lung adenocarcinoma were excluded. The inclusion criteria were as follows: (1) patients diagnosed with lung adenocarcinoma, (2) availability of both transcriptomic and DNA methylation data, (3) presence of patient survival information, and (4) availability of downloadable data for further secondary analysis.

To guarantee the comparability of the cohorts, we used the sva R package and Perl's ComBat normalization technique to co-normalize the data into a unified cohort. The raw data was re-normalized,with the batch correction of DNA methylation microarray data done through the minfi,impute,and wateRmelon R packages [13, 14]. The limma R package was used to perform normal-exponential background correction and between-arrays quantile normalization on the mRNA array outputs. In addition, a weighted linear regression method was used to normalize the expression, and the gene expression values were obtained by multiplying the estimated precision weights of each observation with the corresponding log2 expression values.

A univariate Cox proportional hazards model analysis was conducted on the training cohort, with Bonferroni correction applied for multiple comparisons and a significance threshold of *P* < 0.05, in order to detect prognostic immune signatures. This analysis was performed using the survival R package. From the IMMPORT database(https: //www.immport.org/),immune genes were procured.

We utilized the CIBERSORT algorithm [15] to characterize the immune cell landscape in our study, which permits the quantification and differentiation of 22 human immune cell phenotypes based on transcriptomic data. A thorough evaluation of seven T cell varieties(CD8 T cells,CD4 naive T cells,CD4 memory resting T cells,CD4 memory activated T cells, follicular helper T cells, regulatory T cells, and gamma delta T cells)is encompassed by this comprehensive approach, as well as naive and memory B cells, plasma cells, NK cells, and a variety of myeloid subsets.

### The transposase-accessible chromatin sequencing (ATACseq)

ATAC-seq experiments, we modified the protocol outlined by Daniel [16, 17], isolating M0, M1, and M2 macrophages, and adjusting the number of cells to 50 cells/ml in PBS. The nuclei of these macrophages were then isolated using ATAC-LB. The Agilent Bioanalyzer was employed to evaluate the fragment distribution of the libraries, which were then sequenced on a HiSeq 2500 platform, after the DNA had been broken down and purified, then amplified through 14 cycles.

Our focus was on the ATAC-seq peaks situated near the transcription initiation sites (TSSs) of ZBP1. These peaks were extracted and visualized in a plot showing their distribution in the reads. HOMER and the findMotifsGenome.pl function were employed to analyze the peak regions through motif analysis.

### Isolation of CD14^+^ mononuclear macrophages

Obtaining peripheral blood samples from healthy donors aged 18 to 40 was done by a combination of density gradient centrifugation and differential adhesion. To verify the purity of the isolated mononuclear cells, immunofluorescence staining was done with a CD14^+^ antibody, which serves as a marker for mononuclear cells. The CD14^+^ mononuclear cells obtained from the isolation process were promptly utilized for subsequent experiments.

### Cell culture

The A549 cell line, procured from the Cell Bank of Pricella Life Science & Technology Company (Wuhan, China)in 2022,was utilized for the experiments. Culturing the cells in DMEM(Wisent Biotechnology, Nanjing, China), supplemented with 10%fetal bovine serum(FBS; Coring, Australia),100 IU/ml penicillin, and 100 μg/ml streptomycin, was done at 37° C in a humid atmosphere containing 5%CO2.

### In vitro macrophage polarization

The CD14^+^cells were cultured in Dulbecco's Modified Eagle Medium (DMEM) supplemented with 10%Fetal Bovine Serum(FBS)in order to undergo in vitro macrophage polarization.M1 polarization was achieved by treating cells with 50 ng/mL of Interferons-γ (IFN-γ, Sigma-Aldrich, USA) for 24 h, whereas M2 polarization was achieved by treating cells with 20 ng/mL of Interleukin-4(IL-4,Sigma-Aldrich,USA)for the same duration. The control group consisted of M0-polarized macrophages that were induced with PBS for 24 h. To transition from M1 to M2, the cells were initially stimulated with 50 ng/mL IFN-γ for 24 h. Afterward, there was a modification made to the cell culture medium, and the cells were then washed with PBS. Furthermore, the cells were induced with 20 ng/mL IL-4 for an additional 24 h.

### Plasmids and cell transfection

To begin, we employed PCR to amplify the coding sequence (CDS) region of ZBP1 (NM_030776, 1289 bp). Following this, we utilized the predicted CpG islands from NCBI to identify the methylated CpG islands of cg09897064, which were situated 1100 bp and 1096 bp upstream of the TSS of the ZBP1 transcript within its promoter domain. The promoter regions of the ZBP1 transcript, containing either unmethylated (U) or methylated (M) CpG islands at cg09897064, were subsequently amplified and confirmed by sequencing. Subsequently, we constructed the plasmid by ligating the ZBP1 transcript with the aforementioned promoter fragment. Additional file 1: Table S1 furnishes the initial information. As controls, empty vectors were incorporated into the trial setup.

Furthermore, three custom-designed sequences targeting human ZBP1 were procured from GeneChem Co.com.cn; Additional file 1: Table S2). Lentivirus supernatant was used to transfect CD14 + mononuclear cells at an infection multiplicity of 50. After a three-day transfection period, we conducted a western blot analysis to evaluate the effectiveness of different shZBP1 sequences in down-regulating specific genes.

### Study on mice

The BALB/c mice aged 4–5 weeks, which were supplied by The Servicebio Company in Wuhan, China, were used for all animal experiments, following the regulations of the Institutional Animal Use and Care Committee.

Randomly selecting mice, 4 groups of 6 were formed for the CD14 + mononuclear macrophage experiment. The groups were designated as follows: A549 cells + CD14^+^ cells-ZBP1-U, A549 cells + CD14^+^ cells-shZBP1, A549 cells + CD14^+^ cells-ZBP1-M, and A549 cells + CD14^+^ cells-ZBP1-Ctrl.

For a period of 6 weeks, mice were implanted with a combination of A549 cells (1 × 106) and conditioned macrophages (3 × 106) subcutaneously in their armpits. Subsequently, tumor tissues were collected, weighed, and the M2 macrophage proportion was evaluated.

### Histology and IHC analysis

Tumor tissues were immersed in 4% paraformaldehyde (PFA) solution for an hour, after which they were dehydrated at 4 °C for the night and then embedded in OCT compound. All tissues were cut using an automated instrument according to the manufacturer's instructions to obtain 8-mm sections. Afterwards, the sections underwent a 30%peroxide blocking process, were incubated with mouse monoclonal antibodies against CD206 (ab64693,Abcam), CD86 (ab220188, Abcam) at a 1200 dilution, followed by secondary antibodies(PV-6001,ZSGB-BIO). The color development was performed using a DAB detection kit(ZLI-9018,ZSGB-BIO), followed by hematoxylin counterstaining, differentiation with hydrochloric acid alcohol, and finally mounting of the slides.

### Flow cytometry

The pan-macrophage marker CD68 was used to identify macrophages, while CD80 and CD86 were used as markers for M1 macrophages and CD206 and CD163 were used as markers for M2 macrophages. Fluorescence intensity and cell percentage were utilized to evaluate the abundance of macrophage polarization. The following antibodies were utilized in this study.

CD68: FITC anti-human CD68 mAb#333,805; PE anti-human CD68 mAb# 333,807.

CD86: FITC anti-human CD86 mAb# 374,203.

CD206: FITC anti-human CD206 mAb#321,103; PE anti-human CD206 mAb#321,105.

CD80: PE anti-human CD80 Recombinant#370,611.

CD163: PE anti-human CD163 mAb#333,605.

### Western blot

Western blot analysis was utilized to ascertain the protein concentrations of iNOS and ARG1, with βTubulin as the reference protein. The western blotting procedure followed the methodology described in our previous studies.In this study, the following antibodies were utilized for western blotting.

ZBP1: Cell Signaling Technology, rabbit mAb #60,968;

iNOS: Abcam, rabbit mAb #ab178945;

ARG1: Abcam, rabbit mAb #ab133543;

βTubulin: Abcam, rabbit pAb #ab6046.

### ELISA

Using an enzyme-linked immunosorbent assay (ELISA) kit from Invitrogen(USA),the supernatants of each experimental group were measured for IL1β and IL10 levels, adhering to the manufacturer's instructions. Both methylation-specific PCR and Bisulfite amplicon sequencing (BSAS)are utilized. An examination of the genomic sequence of ZBP1, including a 2 kb area prior to the transcriptional start site (TSS), was conducted. Based on the predicted CpG islands, primers for MSP and BSP were designed and are provided in Additional file 1: Table S1.Genomic DNA from CD14 + mononuclear macrophages was isolated and subjected to bisulfite modification as previously described. Purificationof bisulfite-modified DNA was accomplished with the Wizard DNA Clean Up System(Promega), followed by NaOH(5.5 ml)and ethanol precipitation. Subsequently, the precipitated DNA was re-suspended in water and amplified with either MSP or BSP primers. Afterwards, the DNA fragments were separated on agarose gels containing 2% and made visible through ethidium bromide staining. In order to improve the BSP products, a DNA purification kit from Takara, Dalian was used and inserted into the pGEM-T easy vector by Promega. To assess the methylation status of the ZBP1 sequence, five clones were randomly selected from each cell line and their sequenced data were aligned using Meth BLAST. The primers were shown in Additional file 1: Table S3.

### Luciferase report assay

Transfection of macrophages was performed with luciferase vectors (0.2 mg/well) that were constructed and introduced using Lipofectamine 2000.The negative control consisted of cells transfected with a pGL3 vector lacking a promoter. After a 12-h incubation,the transfected cells in each well were washed twice and then lysed with 100 ml of reporter lysis buffer. The experiment was repeated over three times, with every experimental group having triplicate wells. The dual luciferase activity, expressed in relative light units(RLU),was then determined using a luminometer.

### Electrophoretic mobility shift assays (EMSA)

EMSA experiments were performed to examine the binding between unmethylated and methylated promoter regions of ZBP1 labeled with Biotin at the 5'and 3'ends.A mixture of 0.1 pmol of Biotin-tagged DNA fragments, along with 30 pmol of purified CEBPA,was combined in buffer A(20 mM Hepes, pH 7.5,150 mM NaCl,1.5 mM MgCl2,and 2 mM DTT)supplemented with 2 mM ADPCP. This mixture was then left to incubate at room temperature for 10 min before being analyzed using BisTris native gels (Life Technologies).In EMSA experiments that require a specific duration of time,the reactions were incubated at 37° C for the specified period.

### Clinical validation

We collected lung adenocarcinoma specimens (bronchoscopy forceps or lung puncture samples) from newly diagnosed patients at our respiratory department between December 1st, 2023 and February 1st, 2024. Inclusion criteria: (1) Patients or authorized family members consented to the use of their residual pathological tissue for this study; (2) Diagnosis of lung adenocarcinoma confirmed by immunohistochemistry reports from the hospital pathology department.Exclusion criteria: (1) Presence of autoimmune diseases or immunodeficiency conditions that severely affect the immune microenvironment; (2) Insufficient pathological tissue for further experimentation; (3) Patients on long-term steroid treatment or recent use of immunomodulatory agents. The clinical validation included methylation-specific PCR to detect methylation at the cg09897064 site, Western blot to assess ZBP1 protein expression, and flow cytometry to determine the number of M2 macrophages.

### Statistical analysis

We performed statistical analysis on the data using two-tailed, unpaired Student t-tests, and expressed the results as means ± standard deviation (SD). When* P* values were less than 0.05 differences were deemed statistically significant. For in vivo experiments, each group consisted of six mice, whereas for in vitro experiments, each group consisted of three mice. The histological and western blot analyses were performed three times and consistently showed the same results. For all statistical analyses, R × 643.6 was used.

## Result

### The prognostic importance of cg09897064 methylation on ZBP1 in lung adenocarcinoma

From the TCGA public database, we extracted the methylation and RNA expression matrices, as well as prognostic information of lung adenocarcinoma patients. We conducted univariate Cox regression analysis on the methylation expression and prognostic information of lung cancer patients, with a *P*-value of less than 0.05,to determine prognostic-related genes. Additionally, Pearson correlation analysis was conducted between methylation expression and RNA expression in lung cancer patients, to determine methylation-driven genes, in addition to a total of 497 lung adenocarcinoma cases and 54 adjacent normal tissue samples. Methylation-driven genes were identified whentheir correlation coefficient was greater than 0.3 and their *P*-value was less than 0.05.

**Fig. 1:**
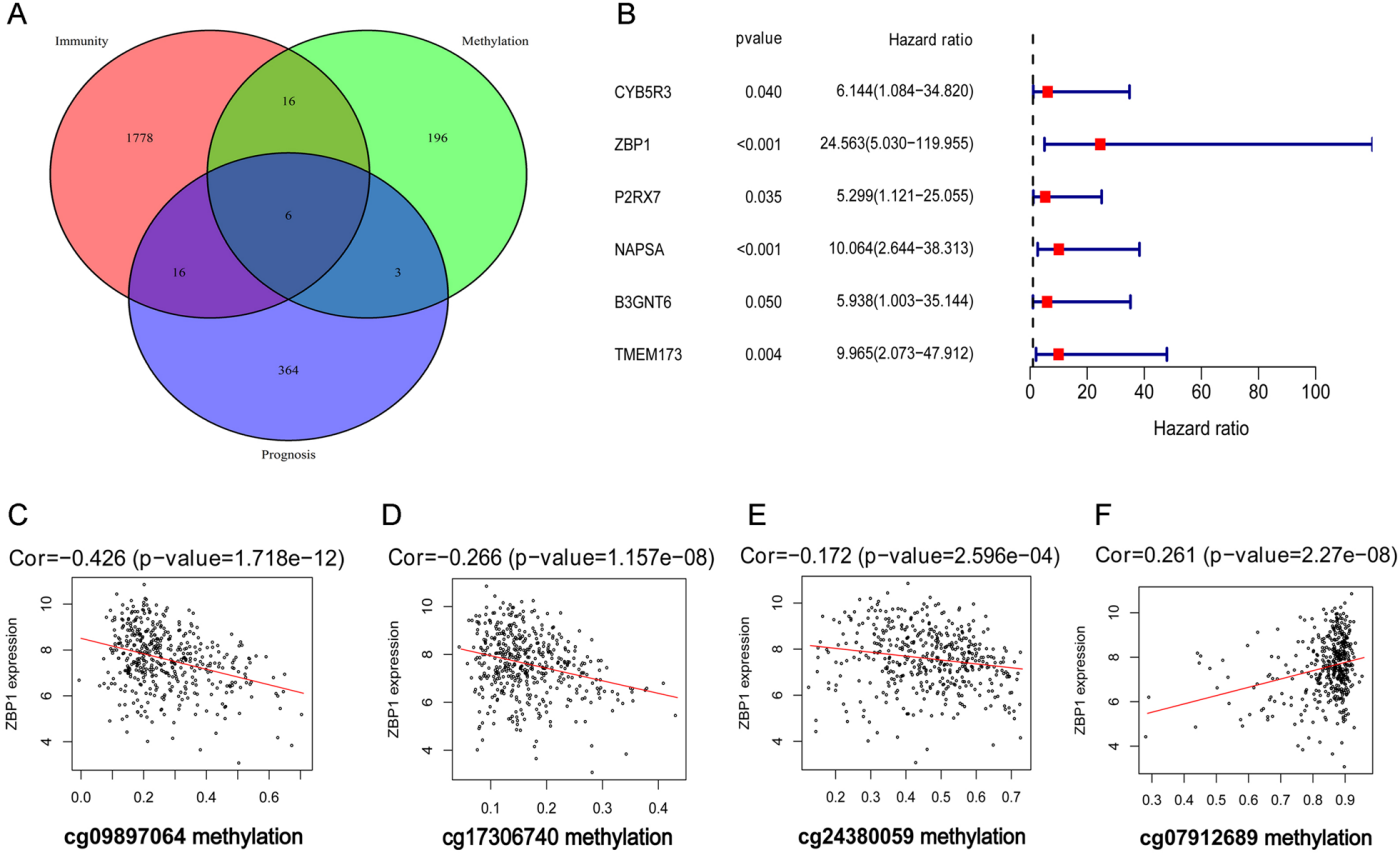
The Prognostic Value of Methylation on cg09897064 in ZBP1 in Lung Adenocarcinoma. The TCGA database was used to extract methylation and RNA expression data, as well as prognostic information of lung adenocarcinoma patients. **A**. Screening of prognostic-related methylation-driven immune genes: 6 overlapping genes were identified as prognostic-related methylation-driven immune genes among 2504 immune genes, 221 methylation driver genes, and 389 prognostic genes. **B**. The univariate COX regression analysis unveiled that the total methylation expression of ZBP1 gene has the most significant impact on prognosis,highlighting methylation as a key determinant of immune gene prognostic information. **C**-**F**. Among the 4 methylation sites,cg09897064 demonstrated the strongest correlation with ZBP1 mRNA expression

Incorporating genes from the IMMPORT database (https://www.org/), we included immune genes. By performing an intersection analysis among the three gene sets, we identified 6 genes that were both prognostic-related, methylation-driven, and immune-related (Fig. 1A). The univariate Cox regression of Fig. 1B reveals the correlation between the methylation expression of these 6 genes and prognosis, with ZBP1 having the most noteworthy effect (highest HR, lowest *P*-value).

ZBP1 is known to have 4 methylation sites: cg07912689, cg09897064, cg17306740, and cg24380059. The correlation analysis (Fig. 1C-F) suggests that cg09897064 methylation has the highest correlation with ZBP1 mRNA expression (highest absolute correlation coefficient of -0.426, lowest *P*-value). Hence, we infer that the methylation of cg09897064 greatly impacts the transcription of ZBP1 mRNA.

### Association between cg09897064 methylation on ZBP1 and lung adenocarcinoma prognosis and macrophage polarization

**Fig. 2:**
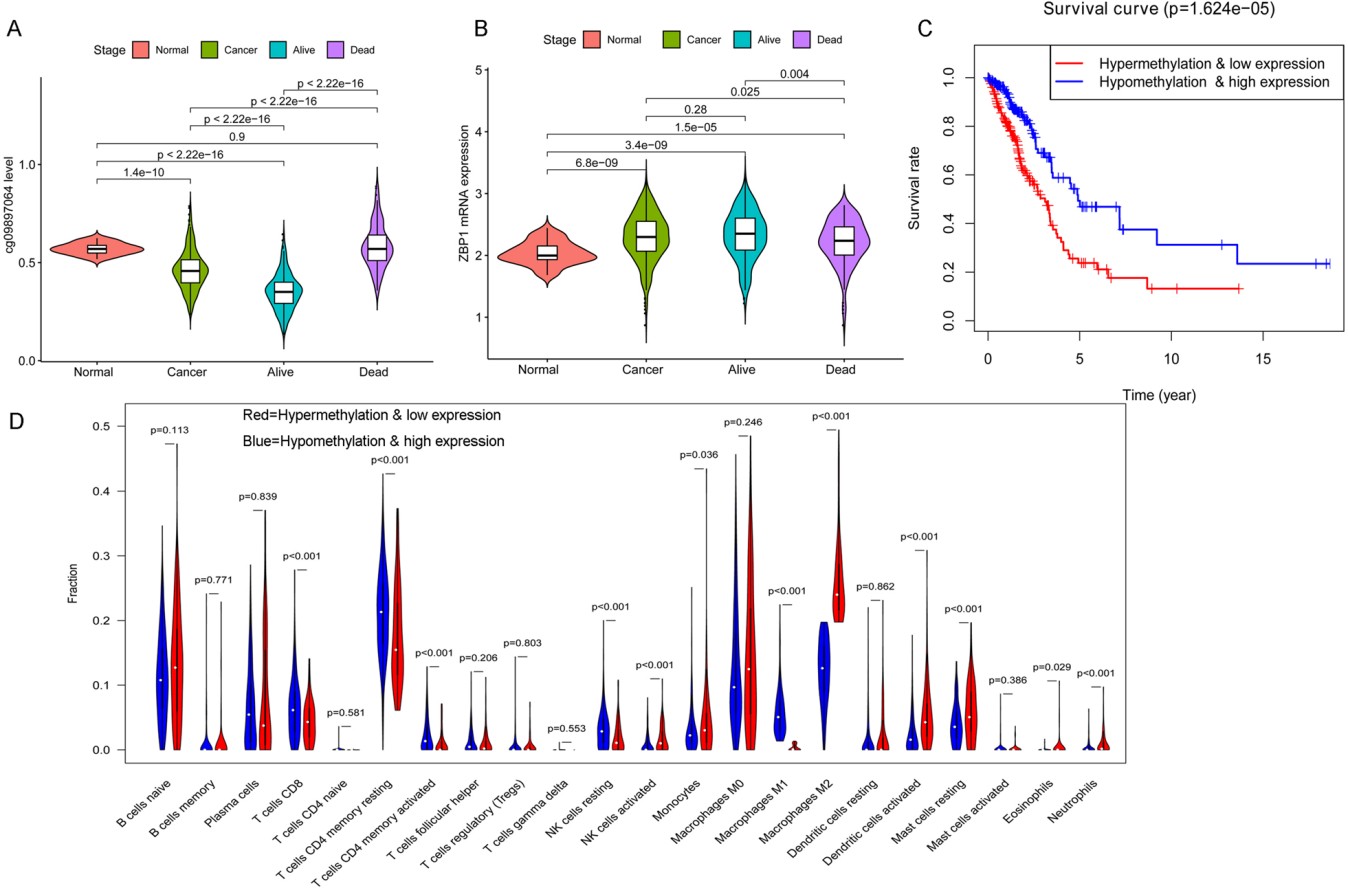
Association between cg09897064 methylation on ZBP1 and lung adenocarcinoma prognosis and macrophage polarization. Further analysis of lung adenocarcinoma data from TCGA database revealed the following findings: **A**. In adjacent tissues, a higher methylation of cg09897064 was seen than in lung adenocarcinoma tissues (*P* < 0.001). **B**. Significantly higher mRNA expression of ZBP1 was observed in lung adenocarcinoma tissues compared to neighboring tissues (*P* < 0.01). **C**. Patients who had low levels of cg09897064 methylation and high levels of ZBP1 mRNA expression had a significantly improved prognosis compared to thosewith high levels of cg09897064 methylation and low levels of ZBP1 mRNA expression (*P* < 0.001). **D**. cg09897064 methylation and tumor immune microenvironment: The "cg09897064 low methylation & ZBP1 mRNA high expression group" showed increased M1 macrophages and decreased M2 macrophages compared to the "cg09897064 high methylation & ZBP1 mRNA low expression group"(*P* < 0.01)

Further analysis of lung adenocarcinoma data from TCGA database revealed the following findings: a comparison of cg09897064 methylation levels in various tissue samples revealed that cg09897064 had a greater methylation in adjacent normal tissue than lung adenocarcinoma tissue (*P* < 0.001). Additionally, lung adenocarcinoma tissue from deceased patients had higher methylation levels of cg09897064 compared to surviving patients (*P* < 0.001), shown in Fig. 2A. The results indicate that the presence of cg09897064 methylation is linked to an unfavorable prognosis among individuals with lung adenocarcinoma.

The expression of ZBP1 mRNA was also examined in different tissue samples. A marked difference in ZBP1 mRNA expression betweenlung adenocarcinoma tissue and its neighboring normal tissue was noted (*P* < 0.001). Furthermore, ZBP1 mRNA expression was significantly higher in lung adenocarcinoma tissue from surviving patients compared to deceased patients(*P* < 0.01),shown in Fig. 2B.The expression of ZBP1 mRNA being low appears to be a risk factor for a dismal outcome in lung adenocarcinoma patients, as these results demonstrate.

Patients were categorized into two groups, namely the "high methylation of cg09897064 and low expression of ZBP1 mRNA group" and the "low methylation of cg09897064 and high expression of ZBP1 mRNA group", based on the median values of cg09897064 methylation level and ZBP1 mRNA expression. Survival analysis of these two groups revealed that patients in the "low methylation of cg09897064& high expression of ZBP1 mRNA group" had a significantly better prognosis compared to those in the "high methylation of cg09897064& low expression of ZBP1 mRNA group" (*P* < 0.001), shown in Fig. 2C.

Furthermore, the impact of cg09897064 methylation on the tumor immune microenvironment was investigated using the "CIBERSORT" algorithm. A comparison of the two groups of lung adenocarcinoma tissue revealed considerable disparities in the proportions of different immune cells (*P* < 0.01). Particularly, the "low methylation of cg09897064& high expression of ZBP1 mRNA group" showed a marked rise in M1 macrophages and a noteworthy decrease in M2 macrophages when compared to the "high methylation of cg09897064& low expression of ZBP1 mRNA group", as depicted in Fig. 2D.

To sum up, the evidence implies that cg09897064 methylation is linked to a lack of ZBP1 expression and encourages the polarization of M2 macrophages. In addition, the methylation of cg09897064 is associated with a negative prognosis in patients with lung adenocarcinoma.

### In vitro induction of ZBP1 down-regulation and macrophage M2 polarization by cg09897064 methylation

To explore the association between cg09897064 methylation and the manifestation of ZBP1 during macrophage polarization, a series of experiments were conducted. CD14^+^ mononuclear macrophages were exposed to IFNγ and IL4, respectively, to provoke M1 and M2 polarization. The CD14^+^ mononuclear macrophages were sourced from Pricella Life Science&Technology Company in Wuhan, China, and confirmed through immunofluorescence staining (Additional file 1: Figure S1).

**Fig. 3:**
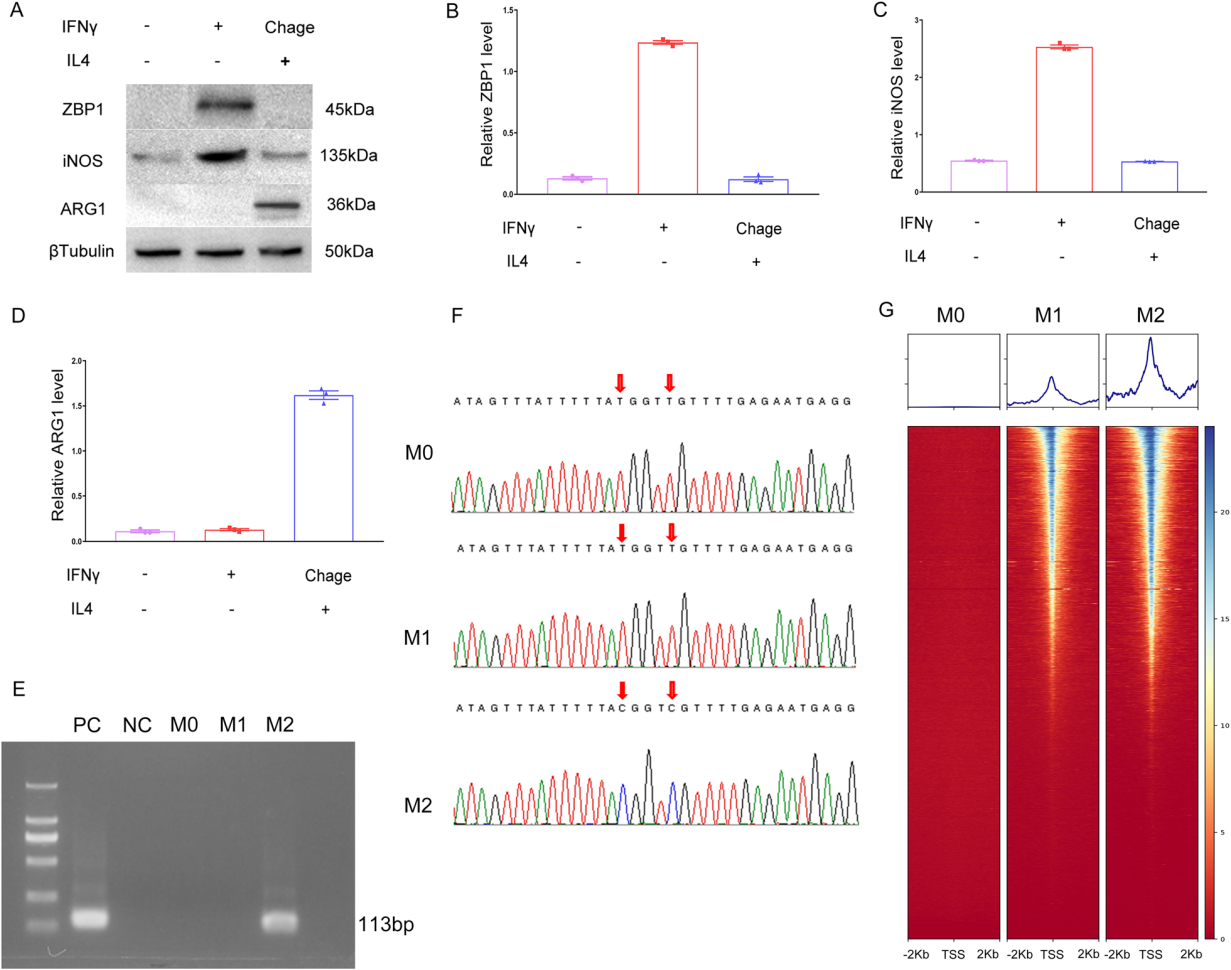
This study examines the effects of cg09897064 methylation on ZBP1 expression and chromatin accessibility during macrophage polarization. **A**-**D**. ZBP1 protein expression: M1 and M2 macrophage polarization models were established by treating CD14^+^ mononuclear macrophages with IFNγ and IL4, respectively. An evaluation of protein expression levels of iNOS (a M1 macrophage marker), ARG1(a M2 macrophage marker), and ZBP1 in M0, M1, and M2 macrophages was conducted via Western blot analysis. The relative levels of iNOS and ARG1 proteins verified the successful polarization of macrophages;ZBP1 was highly expressed in M1 macrophages,while it was found to be low in both M0 and M2 macrophages. **E**–**F**. Employing methylation-specific PCR(MSP)and bisulfite amplicon sequencing(BSAS),the methylation status of cg09897064 in M0,M1,and M2 macrophages was analyzed. **G**. chromatin accessibility: ATACseq heatmap and peak chart visualization were utilized to evaluate the opening or closing of chromatin regions, specifically on ZBP1 and its promoter region, during macrophage polarization. Statistical significance was determined by conducting appropriate tests (*P* < 0.05 for significance: *, *P* < 0.01 for high significance: **, and* P* < 0.001 for very high significance: ***) on the mean ± standard deviation of the provided dat

A successful establishment of an in vitro model for M1 and M2 polarized macrophages demonstrated a significant increase in ZBP1 expression in M1 macrophages, as compared to both M0 and M2 polarized macrophages, as depicted in Fig. 3A-D through Western blot analysis.

To further investigate the link between cg09897064 methylation and ZBP1 expression, we conducted MSP and BSAS. Our research uncovered cg09897064 methylation in M2 macrophage polarization, yet not in M0 and M1 polarized macrophages. This observation may provide an explanation for the decreased expression of ZBP1 in M2 polarized macrophages, shown in Fig. 3E-F.

In the subsequent investigation, we focused on the downregulation of ZBP1 in M0 polarized macrophages. The ATACseq analysis showed that the chromatin accessibility of ZBP1 and its promoter region was closed in M0 polarized macrophages, but open in both M1 and M2 polarized macrophages. This finding suggests that the downregulation of ZBP1 in M0 macrophages may be associated with the differential chromatin accessibility dynamics during macrophage polarization, shown in Fig. 3G.

**Fig. 4:**
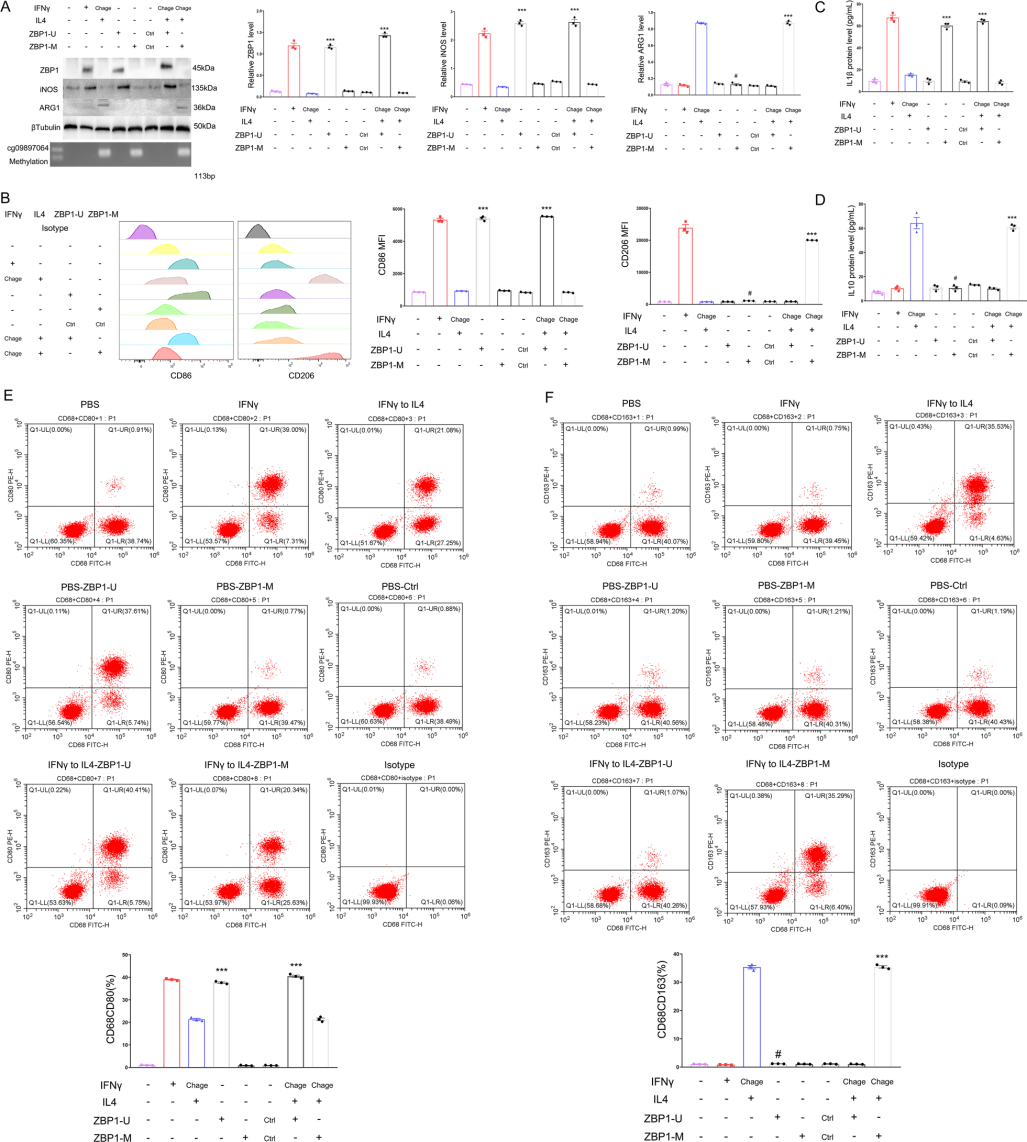
In vitro regulation of ZBP1 down-regulation and macrophage M2 polarization by cg09897064 methylation. CD14^+^ mononuclear macrophages were transfected with the ZBP1-unmethylation overexpression plasmid (ZBP1-U), ZBP1-methylation overexpression plasmid (ZBP1-M), or blank control plasmid (Ctrl). **A**. Protein expression levels of Inos (M1 macrophage marker), ARG1 (M2 macrophage marker), and ZBP1 were assessed by Western blot analysis in M0,M1,and M2 macrophages. **B**. Flow cytometric analysis was conducted to ascertain the expression of CD86 (M1 macrophage marker) and CD206 (M2 macrophage marker) following transfection with either ZBP1-U or ZBP1-M. **C**-**D**. ELISA was used to measure the levels of IL1β (M1 macrophage marker) and IL10(M2 macrophage marker) in the culture medium of CD14^+^ mononuclear macrophages from the experimental groups. **E**–**F**. Flow cytometric analysis was performed to determine the percentage of CD68^+^CD80^+^ (M1 macrophage marker) and CD68^+^CD163^+^ (M2 macrophage marker) after transfection with either ZBP1-U or ZBP1-M. Statistical significance was established through the application of suitable tests(*P < 0.05,**P < 0.01,***P < 0.001)for the mean ± standard deviation data presented

To investigate the impact of cg09897064 methylation and ZBP1 expression on macrophage polarization from M1 to M2,we constructed ZBP1-unmethylation overexpression plasmid (ZBP1-U), ZBP1-methylation overexpression plasmid(cg09897064 methylation) (ZBP1-M), and a blank control plasmid(Ctrl), which were then transfected into mononuclear macrophages. Flow cytometry, ELISA, and Western blotting were performed to evaluate the expression of M1 polarized markers(iNOS,CD86,and IL1) and M2 polarized markers(ARG1,CD206,IL10). Our findings demonstrated that the expression of M1 polarized markers(iNOS,CD86,and IL1)significantly decreased following ZBP1-U transfection,when compared to ZBP1-M or Ctrl plasmids (*P* < 0.001). In Fig. 4,it can be seen that the expression of M2 polarized markers(ARG1,CD206,IL10) was noticeably reduced when comparing macrophage polarization from M1 to M2 after ZBP1-U transfection to cg09897064 methylation on ZBP1(*P* < 0.001).

These findings suggest that ZBP1 inhibits macrophage polarization from M1 to M2, and that cg09897064 methylation suppresses ZBP1 expression, promoting macrophage M2 polarization.

### In vivo regulation of macrophage M2 polarization and mononuclear macrophages-induced tumor growth by cg09897064 methylation

**Fig. 5:**
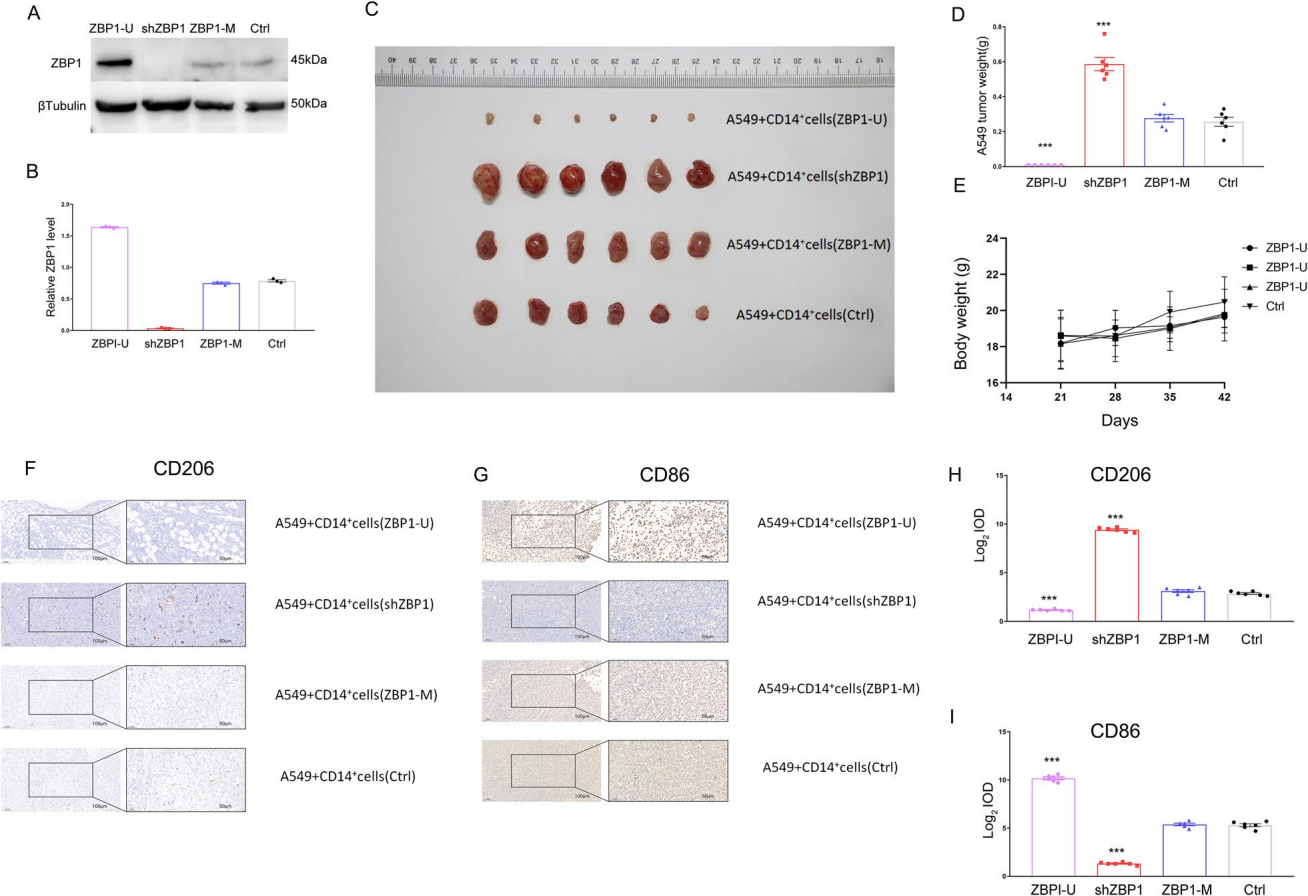
Methylation of cg09897064 regulates the polarization of macrophage M2 in vivo, and the growth of tumors caused by mononuclear macrophages. **A**-**B**. ZBP1 expression. Western blot was performed to assess the ZBP1 levels in tumor tissue from the experimental groups. **C**-**E**. Tumor growth. The tumors formed in mice at the endpoint of mice study.Four groups (n = 6 each) were examined for tumor weight and body weight statistics. **E**–**F**. M1/M2 macrophage polarization. An Image-Pro Plus 6.0 was utilized to analyze the expression scores of CD206 and CD86 in tumor tissues, which had been histopathologically and IHC-impaired, and the macrophage polarization was depicted. The mean ± standard deviation of the presented data was found to have statistical significance using appropriate tests(*P < 0.05,**P < 0.01,***P < 0.001)

An in vivo experiment was conducted to explore the influence of cg09897064 methylation and ZBP1 expression on the interactions between macrophage and tumor cells, particularly in terms of the promotion of tumorigenesis by M2 macrophages. CD14^+^ mononuclear macrophages were infected with ZBP1-U, ZBP1-M, shZBP1, or control lentivirus and mixed with A549 lung carcinoma epithelial cells. Mice had their armpits implanted with the cell mixture subcutaneously for a period of 6 weeks. Lentiviral transduction enabled stable knockdown of ZBP1 in the shZBP1 group, compared to the control virus-transduced cells. Furthermore, the ZBP1-U plasmid significantly upregulated ZBP1 expression compared to the ZBP1-M transduced cells (*P* < 0.001) (Fig. 5A and B).Visible distinctions in the proliferation of primary tumors after subcutaneous injection of the cell mixture were seen between the control group and the shZBP1 group(Figs. 5C and D),with no noteworthy variations in the mice's body weight(Additional file 1: Figure S2 and Figs. 5E).

Analysis of the expression of CD206 was performed using immunohistochemistry(IHC), as demonstrated in Fig. 5F. Results showed that the ZBP1-U expression group had a lower CD206 expression in the tumor region compared to the ZBP1-M expression group, while the shZBP1 group had a higher CD206 expression in the tumor region. This assessment was done to evaluate the possible roles of cg09897064 methylation and ZBP1 expression in macrophage M2 polarization-induced tumor growth. Quantitative analysis using the log IOD in Image-Pro Plus 6.0 further confirmed the correlation between CD206 expression levels and tumor growth (Fig. 5G).

### Impaired CEBPA binding to ZBP1 promoter domain due to cg09897064 methylation

**Fig. 6:**
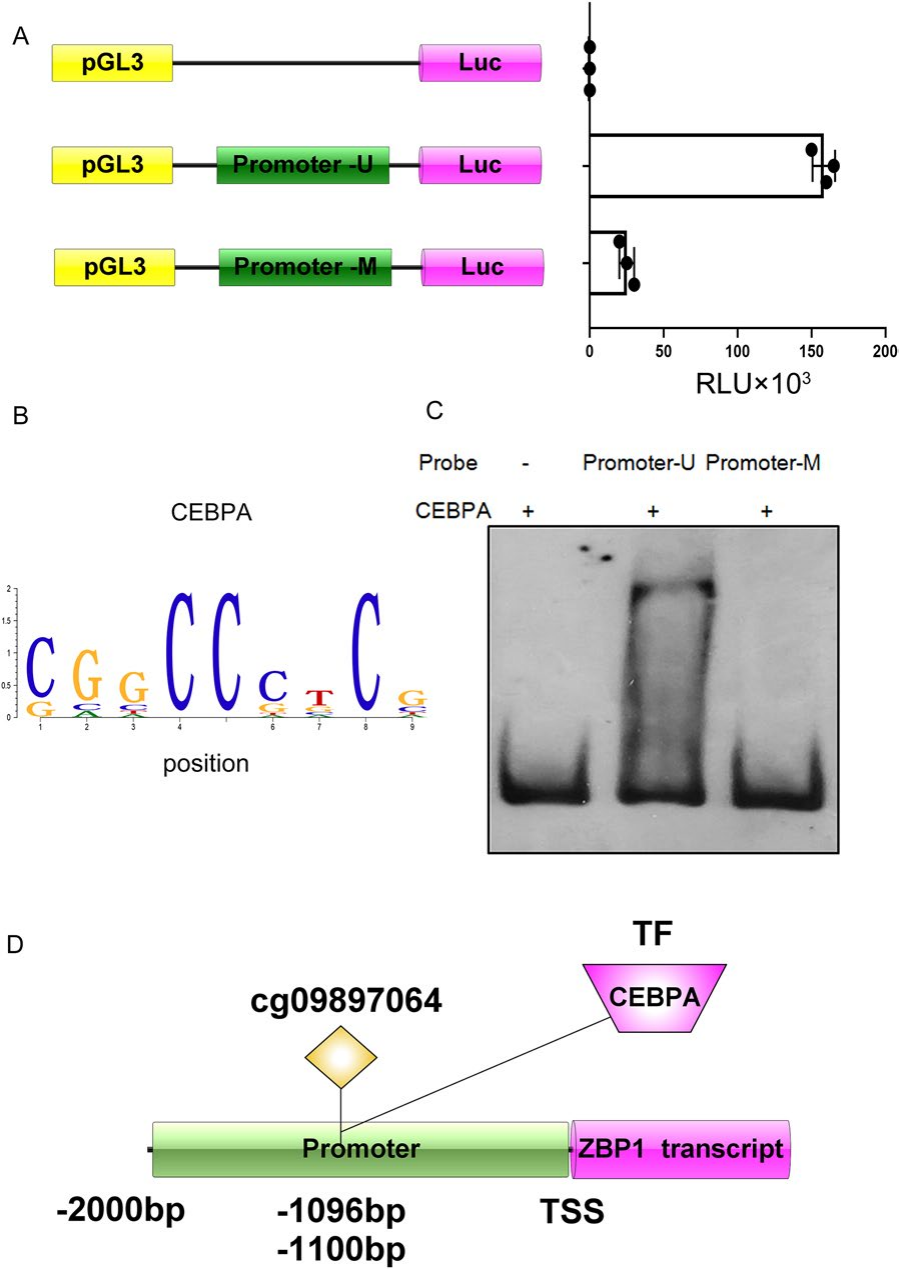
cg09897064 methylation impairs the CEBPA binding to promoter domain of ZBP1. **A**. CD14^+^ mononuclear macrophages, transfected with both the pGL3 luciferase vector and the pGL3 control vector, were washed and subjected to a luciferase activity assay after 24 h.The relative luciferase activity was then measured in triplicate wells, with both unmethylated and methylated promoter regions present. **B**. Motif analysis of ATACseq data revealed that CEBPA binds to the promoter domain of ZBP1, as indicated by the identified binding sites. **C**. An electrophoretic mobility shift assay(EMSA)was employed to evaluate the CEBPA's adhesion to both unmethylated and methylated promoter areas. **D**. The diagram illustrates how cg09897064 methylation regulates ZBP1 transcription. It shows that cg09897064 methylation impairs the binding of CEBPA (Transcription factor, TF) to the promoter domain of ZBP1. Statistical significance was established through appropriate tests(*P < 0.05,**P < 0.01,***P < 0.001)for the mean ± standard deviation of the presented data

We conducted an analysis of the CpG island methylation at the cg09897064 site on ZBP1 using the National Center forBiotechnology Information (NCBI) database in order to understand the mechanism by which cg09897064 hampers ZBP1 expression. We found that the methylated CpG islands of cg09897064 were located upstream of the ZBP1 transcript's TSS at -1100 bp and -1096 bp, within the promoter domain of ZBP1.We hypothesized that methylation of cg09897064 would impede the promoter's transcriptional activity. To verify this, luciferase reporter plasmids were constructed with either unmethylated (U) or methylated (M) regions (Fig. 6A). These plasmids were transfected into CD14 + mononuclear macrophages to assess their transcriptional activity.The pGL3-Promoter-U plasmid, in comparison to the control group and the methylated promoter regions, exhibited a marked rise in luciferase activity (*P* < 0.001), as demonstrated in Fig. 6A.

To further provide solid evidence for this mechanism, we identified CEBPA as the transcription factor (TF) that binds to the CpG island regions through motif analysis of our previous ATACseq data (Fig. 6B). EMSA confirmed that the biotin-labeled promoter-U has the ability to bind to CEBPA, while the promoter-M does not. This is illustrated in Fig. 6C.The methylation of CpG islands in cg09897064 seems to hinder the transcriptional activity of the promoter and also inhibit the binding of CEBPA to the ZBP1 promoter. This conclusion is supported by the findings presented in Fig. 6D.

### Clinical validation of the correlation between methylation at cg09897064, ZBP1 expression, and macrophage M2 polarization

**Fig. 7:**
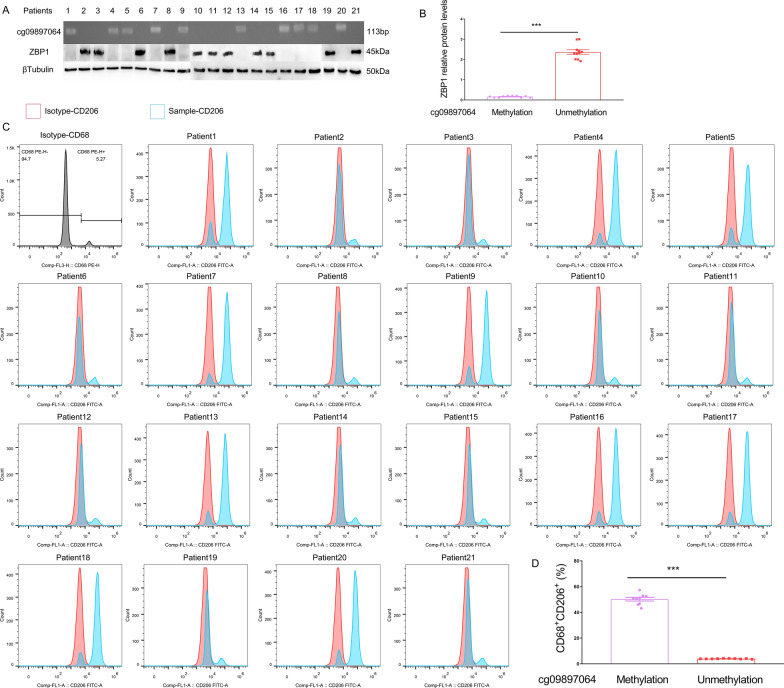
Clinical Validation of the Correlation between cg09897064 Methylation, ZBP1 Expression, and Macrophage M2 Polarization. Twenty-one patients with lung adenocarcinoma were enrolled to assess the correlation between cg09897064 methylation, ZBP1 expression, and macrophage M2 polarization using Methylation-specific PCR (MSP), Western blot, and flow cytometry analysis. **A**/**B**. MSP was performed to detect cg09897064 site methylation in the patients' adenocarcinoma pathological tissues, while Western blot was utilized to measure ZBP1 protein expression. The results showed that 47.6% (10/21) of the adenocarcinoma patients' pathological tissues exhibited methylation at the cg09897064 site. Furthermore, Western blot analysis revealed significantly reduced expression of ZBP1 protein in patients with methylation at the cg09897064 site (*P* < 0.001). **C**/**D**. Flow cytometry analysis was conducted to evaluate the proportion of CD68^+^CD206^+^ cells (M2-polarized macrophages) in single-cell suspensions derived from the adenocarcinoma tissues. The findings demonstrated a significant increase in the proportion of M2 macrophages in the adenocarcinoma tissues of patients with cg09897064 site methylation compared to non-methylated patients (*P* < 0.001)

A total of 21 patients with lung adenocarcinoma were included, and the basic information of the patients is shown in Additional file 1: Table S4. Methylation-specific PCR (MSP) revealed the presence of cg09897064 site methylation in 47.6% (10/21) of the adenocarcinoma patients' pathological tissues. Further Western blot analysis showed significantly reduced expression of ZBP1 protein in patients with methylation at the cg09897064 site, *P* < 0.001 (Fig. 7). Flow cytometry analysis indicated a significant increase in the proportion of M2 macrophages in the adenocarcinoma tissues of patients with cg09897064 site methylation compared to non-methylated patients, *P* < 0.001 (Fig. 7).

## Discussion

Despite significant progress in treatment, lung cancer is still a highly unfavorable disease, with recurrent recurrence and drug resistance. Therefore, it is essential to expand our knowledge of the root cause of lung cancer and discover new therapeutic targets. Adenocarcinoma is highly prevalent in clinical settings, establishing it as the most common malignant tumor and one of the most widespread. Our research has unveiled that cg09897064 methylation and reduced expression of ZBP1 serve as adverse prognostic factors in lung cancer patients. The results of subsequent experiments have been convincing, demonstrating the mechanistic part they play in the transformation of macrophages from an M1 to M2 phenotype, thus promoting tumor progression through M2 macrophages. These discoveries present promising prospects for the creation of novel checkpoint inhibitors targeting lung cancer.

Evidence has been steadily increasing that macrophage polarization is a major factor in the commencement and advancement of tumors [18–20]. Macrophages are a highly plastic cell population that can polarize into distinct functional states depending on the tumor background and disease stage. It is important to recognize that various factors, including cellular and non-cellular components of the immune microenvironment, can influence macrophage polarization in the tumor context.

Immune cells and non-cellular factors within the tumor microenvironment play a critical role in regulating the polarization state of macrophages. Immune cells such as lymphocytes and dendritic cells can induce specific polarization states in macrophages through the secretion of cytokines and stimulation molecules. Furthermore, tumor cells and non-cellular components, such as tumor-secreted cytokines, growth factors, and tumor-associated matrix components, can directly or indirectly impact macrophage polarization. Modulation of these factors can lead to changes in macrophage phenotype and function, thereby influencing tumor growth, invasion, and treatment response.

The polarization state of macrophages is crucial for tumor development and therapeutic responses. In early disease stages, an increase in M1 macrophages can inhibit tumor growth through the release of inflammatory mediators and pro-inflammatory cytokines. However, in late-stage disease, the tumor microenvironment can promote the increase of M2 macrophages, which exhibit immune-suppressive activity and contribute to tumor progression, angiogenesis, and metastasis. Therefore, modulating the polarization state of macrophages may be an important strategy for cancer therapy [21–24].

ZBP1 is a protein which identifies and interacts with Z-DNA, a particular structure of DNA. It regulates gene expression, repairs DNA damage, and contributes to immune response against viral infections.ZBP1 has significant involvement in multiple cellular processes, and its therapeutic potential may be uncovered through additional research [25–28].Our study enhances current understanding, offering new insights into the mechanisms behind macrophage polarization and its impact on tumor growth.In particular,we have determined that ZBP1 plays a crucial role in controlling M1 macrophage polarization. ZBP1, also known as Z-DNA binding protein 1, has been found to promote M1 polarization by activating pro-inflammatory signaling pathways. Through a series of in vitro and in vivo experiments, we have demonstrated that ZBP1 overexpression enhances M1 polarization and subsequently suppresses tumor growth.

Furthermore, our study has revealed a novel epigenetic mechanism involving cg09897064, which acts as a negative regulator of ZBP1 expression. We have observed that cg09897064 methylation leads to the downregulation of ZBP1, consequently promoting M2 polarization of macrophages. The increased tumor growth and progression are linked to the transition to an M2 phenotype. Our findings ultimately enhance the understanding of the complex association between macrophage polarization and tumor growth. By elucidating the molecular mechanisms involved, particularly the role of ZBP1 and cg09897064, our research provides potential targets for therapeutic interventions aimed at modulating macrophage polarization and inhibiting tumor growth.

Furthermore, we conducted clinical validation based on pathological specimens from 21 patients with lung adenocarcinoma and found that cg09897064 methylation is associated with ZBP1 expression and macrophage polarization, representing a novel immune checkpoint. Future studies with larger sample sizes are needed to further confirm the clinical value of cg09897064 methylation as an immune checkpoint in lung adenocarcinoma.

## Conclusion

Our research has identified cg09897064 methylation and reduced expression of ZBP1 as adverse prognostic factors in lung cancer patients. Specifically, we have discovered that ZBP1 promotes M1 polarization, while cg09897064 inhibits ZBP1 expression, leading to M2 polarization and tumor progression. These discoveries offer promising possibilities for the creation of groundbreaking checkpoint inhibitors for the treatment of lung cancer.

### Supplementary Information

**Additional file 1: Table S3.** PCR primers. **Table S4.** Patients’ basic information. **Figure S1.** The immunofluorescence staining for validation of CD14^+^ cell. **Figure S2.** The body weight of the each mice. **Figure S3.** The Ethics approval and consent for investigation on human CD14^+^ cells. **Figure S4.** The Ethics approval and consent for animal experiments.

## Data Availability

The TCGA and GEO database contain all the data produced and/or scrutinized during this research. The study's series in GEO was identified as GSE245440.

## Abbreviations

ZBP1: Z-DNA binding protein 1
CEBPA: CCAAT Enhancer Binding Protein Alpha
TCGA: The Cancer Genome Atlas database
ATACseq: Transposase-Accessible Chromatin Assay
PBS: A phosphate buffer saline solution
TSS: Transcription initiation sites
DMEM: Dulbecco's Modified Eagle Medium
M: Methylated
U: Unmethylated
iNOS: Inducible nitric oxide synthase
IL1β: Interleukin 1β
ELISA: Enzyme-linked immunosorbent assay
MSP: Methylation-specific PCR
BSAS: Bisulfite amplicon sequencing
RLU: Relative light units
EMSA: Electrophoretic mobility shift assays
SD: Standard deviation
IHC: Immunohistochemistry
NCBI: National Center for Biotechnology Information
TF: Transcription factor
