# Supplementary material for: The innovative checkpoint inhibitors of lung adenocarcinoma, cg09897064 methylation and ZBP1 expression reduction, have implications for macrophage polarization and tumor growth in lung cancer
Source: J Transl Med. 2024 Feb 18;22:173. doi: 10.1186/s12967-024-04995-1 (PMC10874569; doi:10.1186/s12967-024-04995-1)
Supplement: Supplementary file 1 — Additional file 1: Table S3. PCR primers. Table S4. Patients’ basic information. Figure S1. The immunofluorescence staining for validation of CD14+ cell. Figure S2. The body weight of the each mice. Figure S3. The Ethics approval and consent for investigation on human CD14+ cells. Figure S4. The Ethics approval and consent for animal experiments. [file 12967_2024_4995_MOESM1_ESM.docx]

Table S1

| ZNP1 | |
| --- | --- |
| Xhol-ZBP1-F: | ATACTCGAGCG-ATGGCCCAGGCTCCTGCTGACCCGG |
| Notl-ANKRD22-R: | ATAGCGGCCGC- CCAAGCCCCACGTGAGGCTGTGCAC |
| Promoter-U | |
| cctctgggtg ggggcaaagg gtagggatgg ggagtggggg gattctgccc tccaagggga  aagggttgct tccagacccc cacagtccac ctttaCGgc**C G**tcctgagaa tgaggacacc  tggatcaaag ctctcctgat ttccctgtac tctgatactt tctacctcat tttaaagttt aatttaattt ttatttttct a | |
| Promoter-M | |
| cctctgggtg ggggcaaagg gtagggatgg ggagtggggg gattctgccc tccaagggga  aagggttgct tccagacccc cacagtccac ctttaCmGgcCm Gtcctgagaa tgaggacacc  tggatcaaag ctctcctgat ttccctgtac tctgatactt tctacctcat tttaaagttt aatttaattt ttatttttct a | |

Table S2

| **shZNP1-1** | |
| --- | --- |
| **top** | **CACCGGGCGGGACTGATCCTGAAGGCGAACCTTCAGGATCAGTCCCGCCC** |
| **bottom** | **AAAAGGGCGGGACTGATCCTGAAGGTTCGCCTTCAGGATCAGTCCCGCCC** |
| shZNP1-2 | |
| top | CACCGCAACATGCAGCTACAATTCCCGAAGGAATTGTAGCTGCATGTTGC |
| bottom | AAAAGCAACATGCAGCTACAATTCCTTCGGGAATTGTAGCTGCATGTTGC |
| shZNP1-3 | |
| top | CACCGCAGGCACCTTCTGGACATGGCGAACCATGTCCAGAAGGTGCCTGC |
| bottom | AAAAGCAGGCACCTTCTGGACATGGTTCGCCATGTCCAGAAGGTGCCTGC |

Table S3 PCR primers

| cg09897064 Methylation | |
| --- | --- |
| F | GGGTGGGGGTAAAGGGTA |
| R | CCTCATTCTCAAAACGACCG |

Table S4 Patients’ basic information

| Patient | ID | Age | Male | TNM stage |
| --- | --- | --- | --- | --- |
| 1 | 308253 | 78 | Male | T3N2M1c，IVB |
| 2 | 435283 | 69 | Female | T2bN3M1c,IVB |
| 3 | 436191 | 71 | Female | T3N2M1a，IVA |
| 4 | 434326 | 46 | Female | T4N3M1c，IVB |
| 5 | 321257 | 62 | Male | T3N2M1c，IVB |
| 6 | 436179 | 69 | Male | T1aN0M0，IA1 |
| 7 | 439347 | 73 | Female | T2N3M1c，IVB |
| 8 | 439087 | 66 | Female | T1bN2M1c，IVB |
| 9 | 441176 | 62 | Female | T4N3M1c，IVB |
| 10 | 408443 | 88 | Female | T3N2M1a，IVA |
| 11 | 245271 | 69 | Female | T4N0M1a，IVA |
| 12 | 439234 | 59 | Male | T1aN0M0，IA1 |
| 13 | 442409 | 60 | Female | T2bN2M1a，IVA |
| 14 | 394460 | 62 | Male | T1bN0M0，IA2 |
| 15 | 039407 | 77 | Female | T1bN0M0，IA2 |
| 16 | 447088 | 71 | Male | T4N3M1c，IVB |
| 17 | 447612 | 75 | Female | T4N3M1c，IVB |
| 18 | 447325 | 82 | Male | T4N2M1b，IVA |
| 19 | 447231 | 75 | Female | T4N0M0，IIIA |
| 20 | 448993 | 52 | Female | T3N2M1b，IVA |
| 21 | 106259 | 63 | Male | T1bN0M0，IA |


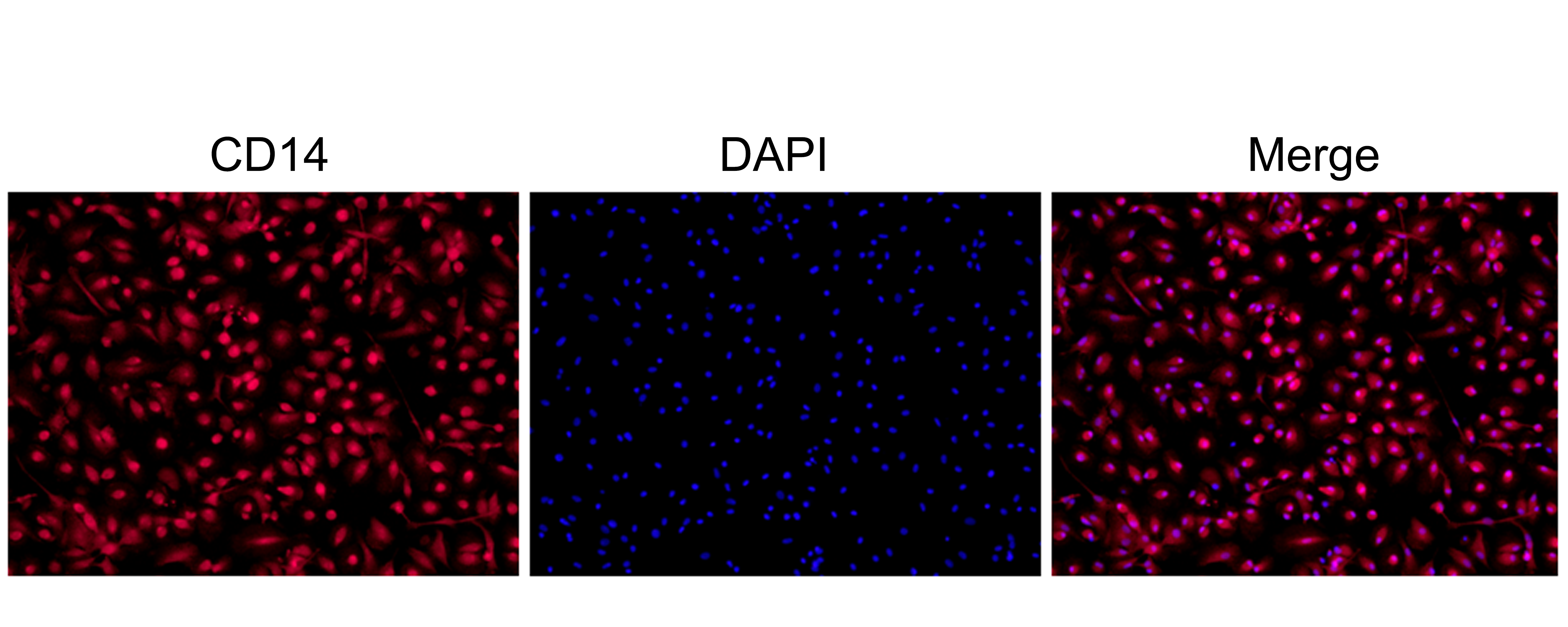


Figure S1. The immunofluorescence staining for validation of CD14^+^ cell


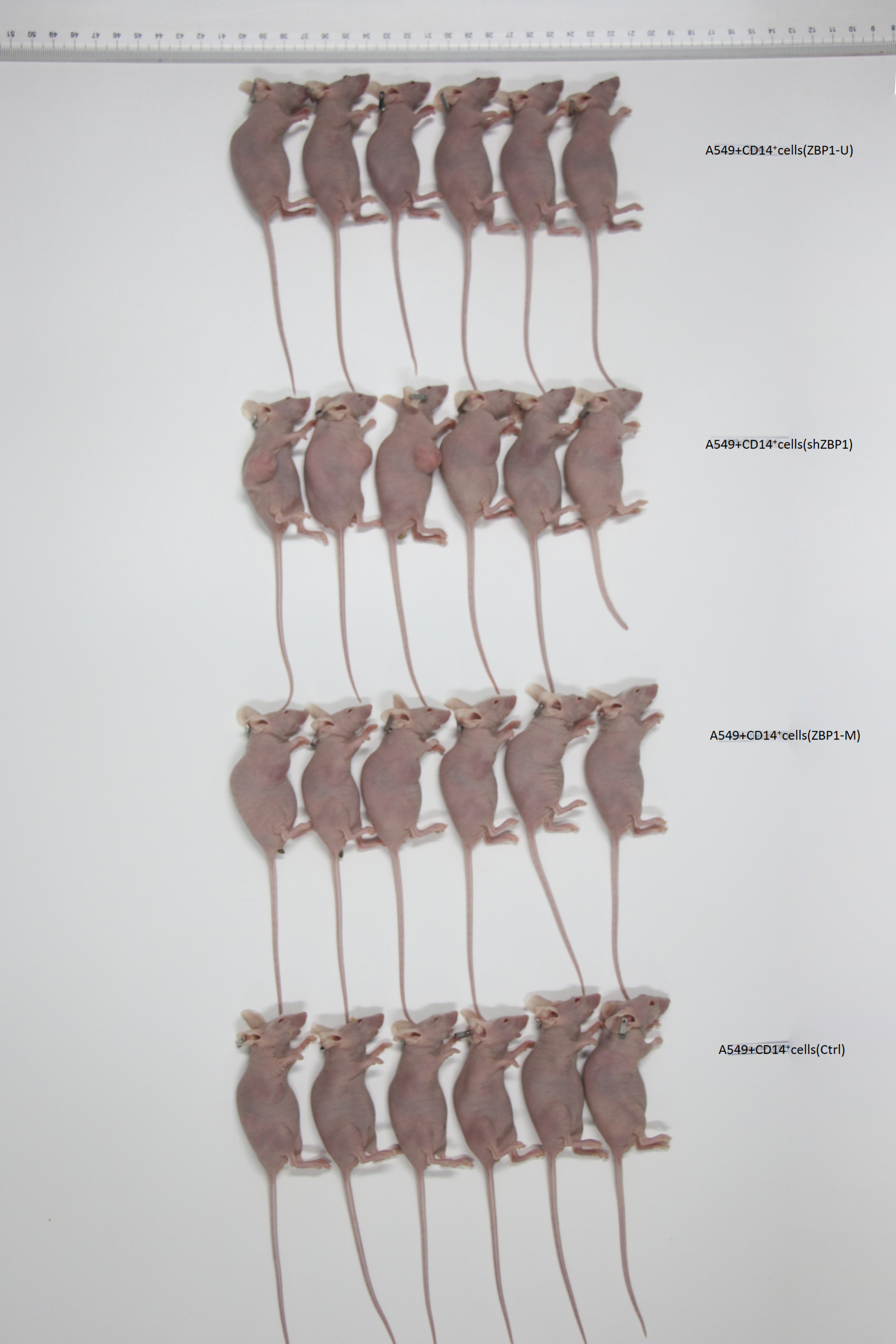


Figure S2. The body weight of the each mice


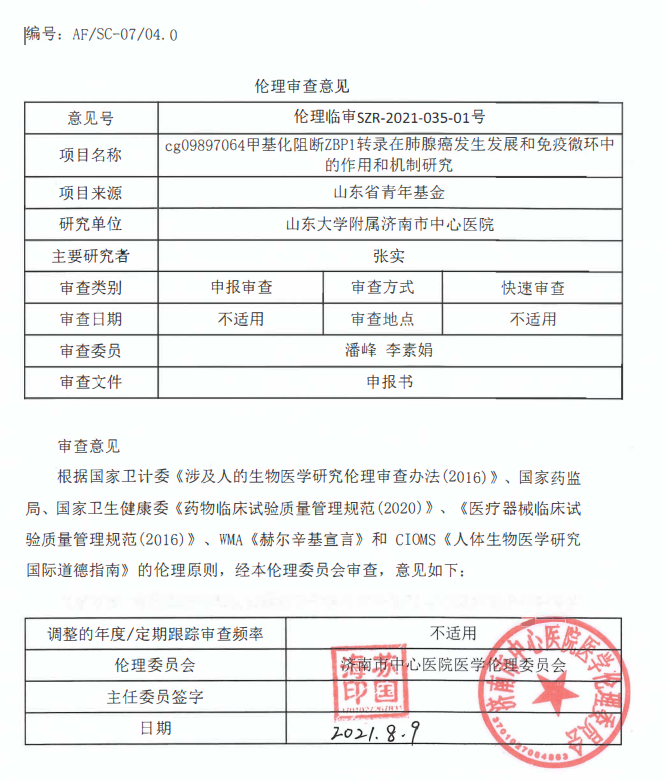


Figure S3. The Ethics approval and consent for investigation on human CD14^+^ cells


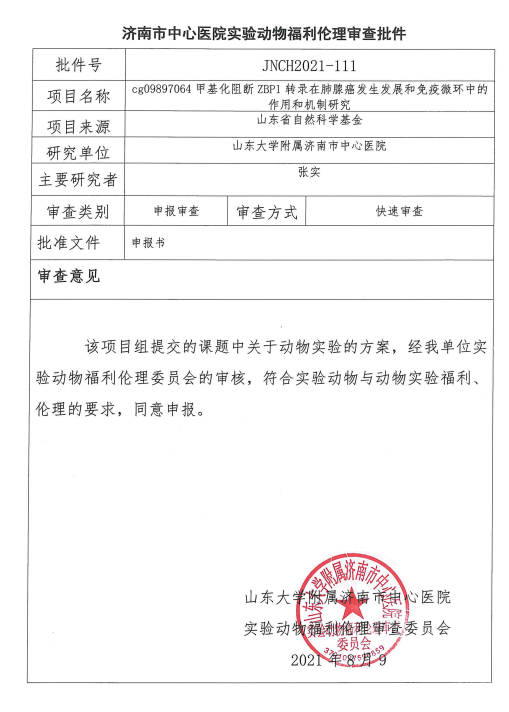


Figure S4. The Ethics approval and consent for animal experiments
